# DeepSEA: an alignment-free explainable approach to annotate antimicrobial resistance proteins

**DOI:** 10.1186/s12859-025-06256-4

**Published:** 2025-09-01

**Authors:** Tiago Cabral Borelli, Alexandre Rossi Paschoal, Ricardo Roberto da Silva

**Affiliations:** 1 Computational Chemical Biology Laboratory, Department of BioMolecular Sciences, School of Pharmaceutical Sciences of Ribeirão Preto, University of São Paulo, Ribeirão Preto, 14040-900 Brazil; 2 NPPNS, Department of BioMolecular Sciences, School of Pharmaceutical Sciences of Ribeirão Preto, University of São Paulo, Ribeirão Preto, 14040-900 Brazil; 3 Cellular and Molecular Biology Program, Department of Cellular and Molecular Biology of Ribeirão Preto, School of Medicine, University of São Paulo, Ribeirão Preto, 14049-900 Brazil; 4 Bioinformatics and Pattern Recognition Group (Bioinfo-CP), Department of Computer Science (DACOM), The Federal University of Technology – Paraná (UTFPR), Cornélio Procópio, Brazil; 5 Rosalind Franklin Institute, Harwell Science and Innovation Campus, Didcot, OX11 0QS UK

**Keywords:** Antimicrobial resistance, Deep learning, Protein annotation, Model explainability

## Abstract

Antimicrobial resistance (AMR) is one of the most concerning modern threats as it places a greater burden on health systems than HIV and malaria combined. Current surveillance strategies for tracking antimicrobial resistance (AMR) rely on genomic comparisons and depend on sequence alignment with strict similarity cutoffs of greater than 95%. Therefore, these methods have high false-negative error rates due to a lack of reference sequences with a representative coverage of AMR protein diversity. Deep learning has been used as an alternative to sequence alignment, as artificial neural networks can extract abstract features from data, thereby limiting the need for sequence comparisons. Here, a convolutional neural network (CNN) was trained to differentiate between antimicrobial resistance proteins and non-resistance proteins, and to annotate them in nine resistance classes. Our model demonstrated higher recall values (> 0.9) than the alignment-based approach for all protein classes tested. Additionally, our CNN architecture allowed us to investigate internal states and explain the model classification regarding protein domain feature importance related to antimicrobial molecule inactivation. Finally, we built an open-source bioinformatic tool (https://github.com/computational-chemical-biology/DeepSEA-project) that can be used to annotate antimicrobial resistance proteins and provide information on protein domains without sequence alignment.

**Supplementary Information:**

The online version contains supplementary material available at 10.1186/s12859-025-06256-4.

## Background

Starting with penicillin [11], antimicrobials enabled efficient treatment of bacterial infections that cause dangerous diseases such as syphilis and gonorrhea. More recently, antimicrobials have allowed patients to undergo invasive or immunosuppressive procedures, which has promoted a substantial increase in human life expectancy [45]. However, as (i) antimicrobials are natural products of microorganisms’ metabolism and (ii) they are employed in competition, antimicrobial resistance is a natural adaptation in the microorganisms’ evolution [37]. As humankind started to use antimicrobials broadly, we increased the selective pressure on bacteria and, consequently, selected resistant variants that need increasingly stronger doses to be contained. This positive-feedback loop continued over the past century until it became what has been called the Antibiotic Crisis [29, 34, 42], a global problem that, by the year of 2019, took 1.27 million lives and involved 4.5 million deaths in total [28].

Antimicrobial resistance is increasing, and the last line of antimicrobials, such as colistin, has begun to fail [25]. Therefore, there is a need for developing comprehensive surveillance strategies to track AMR and new drugs to fight resistant bacteria [9]. The current approach is based on sequence alignment to identify resistance genes and proteins. In this alignment-based model, *in silico* functional annotation is inferred by the “excess” of homology between a candidate and known sequence [32]. A candidate sequence is considered resistant if it shares similarity to the reference sequence above a given threshold [1, 10]. Dedicated databases and tools based on antimicrobial susceptibility tests [14] have different cutoffs to annotate query sequences as resistant proteins. For instance, Resistance Gene Identifier (RGI) [1] uses curated bitscore values of each reference protein stored in its database, whereas AMRFinderPlus [10] and ResFinder [12] use raw similarity and coverage values.

The dependency on reference sequences for alignment-based models constrains surveillance to rediscover the same proteins/genes and to have a high number of false negatives (resistant proteins classified as non-resistant) [2], as most tools use strict cutoffs above 80% [12] or 95% [30] of similarity to annotate a protein as resistant. Deep learning-based models have emerged as a solution to this issue, as neural networks can learn complex and non-linear rules from data and use them to annotate proteins without direct sequence alignment to known references [4]. In this regard, the pioneers DeepARG [2] and HMD-ARG [22] achieved remarkable results in multiclass task classification of resistance proteins with lower false negative rates than alignment-based alternatives. However, these tools either need protein alignment in some stages of their workflow or need a complex approach to make the output explainable.

In this work, we present a study on the ability of a convolutional neural network (CNN) to annotate resistance proteins. Our CNN model had an equivalent performance to the cutting-edge protein language model from the Evolutionary Scale® (https://www.evolutionaryscale.ai/) and outperformed the alignment-based approach. The CNN was able to classify proteins across nine classes and differentiate them from non-resistance proteins with recall (true positives / relevant elements) values above 0.95. We also presented an algorithm to process the CNN’s neuron firing patterns that explain CNN decision-making. Finally, we wrap the model and the algorithm in a command-line interface tool to provide insightful outputs for users with a biological background.

## Methods

### Database construction

For model training, hyperparameter optimization, and evaluation, we selected the Non-redundant Comprehensive Database (NCRD) [27]. The NCRD was built to provide more reference sequences for alignment-based annotation of antimicrobial proteins. The NCRD developers collected consolidated resistance proteins from the Antibiotic Resistance Genes Database (ARDB) [24], Comprehensive Antibiotic Resistance Database (CARD) [1], and Structured Antibiotic Resistance Genes (SARG) [44] and expanded the database using DIAMOND [8] (with the parameters E-value ≤ 1×10-5, Query Coverage HSP ≥ 90%, and Percentage of Positive Positions ≥ 90%) to find homologous proteins from Non-redundant Protein Database (https://www.ncbi.nlm.nih.gov/refseq/about/nonredundantproteins/) and Protein Bank Database (https://www.rcsb.org/). For this work, we selected the NCRD95 (downloaded December 12, 2023), a database version in which the sequence similarity was limited to 95% [21].

The NCRD95 was converted from FASTA to table format via SeqKit 2.8.1 [36], and the FASTA identifiers were inspected to retrieve information on the content of antimicrobial resistance protein classes. Cumulative class curves were employed to (i) determine the optimal number of protein resistance classes that minimize data imbalance while maximizing the database size and (ii) eliminate less representative protein subclasses within each class.

To build the non-resistance class (NonR), we downloaded 191,535 curated bacterial proteins from SwissProt (downloaded January 10, 2023) that are not associated with antimicrobial resistance [2], [22], [43]. The search was made with the query *(taxonomy_id:2) NOT (keyword: KW-0046)* on the Uniprot website, and only the reviewed subset of proteins was downloaded. Then, CD-HIT version 4.8.1 was applied to limit protein similarity to 95%. Finally, DIAMOND version 2.0.8 (with coverage >80%, E-value ≤ 1×10-3, and max-target-seqs = 1) was used to align SwissProt against NCRD95 proteins. Approximately 4600 SwissProt proteins significantly aligned to NCRD95 despite not being associated with antimicrobial resistance and, therefore, could not be used as the NonR class. Using the remaining SwissProt proteins as the NonR class would hugely increase the dataset imbalance,therefore, we randomly selected approximately 4600 proteins from the SwissProt subset that did not align to NCRD95. Therefore, the NonR class is composed of proteins with no relevant sequence similarity to antimicrobial resistance proteins in our dataset.

### Model architecture, training, and evaluation

We used the *train_test_split* function from scikit-learn version 1.4.2 to split the final dataset into training and holdout test sets at an 80/20 ratio, stratified according to the original protein class distribution. The training set was used for hyperparameter (Table 1) optimization with the Keras Hyperband (https://keras.io/keras_tuner/api/tuners/hyperband/) heuristic algorithm and 5-fold cross-validation (five random stratified split points). The hold-out test was used for model evaluation and benchmarking. For reproducibility, the *random_state* (seed) parameter was set to 42 for all split steps performed in this work.

**Table 1:** Hyperparameter space

Hyperparameter	Options	Step
Embedding dimension output	from 50 to 200 dimensions	50
Conv1D kernels	from 128 to 1024 kernels	56
Conv1D kernel size	from 3 to 9	2
Learning rate	1e^−3^ and 1e^−5^	–

To establish a baseline for benchmarking, we used Transformers 4.49 [41] and PyTorch 2.6 [31] to fine-tune an Evolutionary Scale Model 2 (ESM2) [23] from a protein language model of general-purpose to an antimicrobial resistance protein classifier. Additionally, RGI version 6.0.3 and AMRFinderPlus 4.0.19 outputs were manually inspected and mapped from antimicrobial molecule names to broader classes, and results below their respective thresholds were considered Non-resistant proteins.

We used TensorFlow 2.15 and Keras 2.15 to design an end-to-end convolutional neural network model that received input batches of raw amino acid sequences instead of multiple sequence alignment files. Our architecture contains 3 blocks: (i) a data processing block that contains a text2vec as the input layer and a subsequent embedding layer, (ii) a feature extraction block composed of four 1D convolutional layers interpolated with dropout layers used for regularization, and (iii) a classification block with global average pooling and dense layers.

Reference proteins from the same classes were retrieved from the reference catalog of the National Database of Antibiotic-Resistant Organisms (NDARO [downloaded March 12, 2025]) and used to evaluate our model on an independent dataset. The DIAMOND (E-value ≤ 1×10–3, and max-target-seqs = 1 and coverage = 100%) was used to align them against our training set to reveal the distribution.

### Feature extraction

Algorithm 1 describes how we access the matrices within our CNN and process them (Additional File 1; Supplementary Figure 1). First of all, a protein sequence is converted to tensor format to fit input requirements (line 2), and then the CNN model is applied (line 4) to create a probability distribution from which the most probable class index is retrieved (line 6). The dense layer contains weight vectors that convert the outputs from the upstream layer into a probability distribution according to the number of classes in the training set and TensorFlow/Keras frameworks allow access and manipulation of these weight vectors. Therefore, in lines 8 and 9, the algorithm retrieves the weight vector for the class predicted by the model. In line 11, a submodel is temporarily created by removing the global average pooling and dense layers from our CNN. This new model’s output is a matrix with features extracted by convolution. The protein sequence is used by the submodel to obtain and retrieve the feature matrix (lines 13 and 14). Finally, the feature matrix and the weight vector are multiplied (line 16) to highlight the most important features in a normalized (line 18) vector with the same length as the imputed protein.

To check if the final weight vector matches the biological properties of the resistance proteins, we combined Algorithm 1 and multiple sequence alignments of enzymes from our holdout test set. For visual clarity, we first clustered enzymes by sequence similarity (up to 90%) and minimal coverage equal to 90%, and the largest cluster of each protein class was selected to be aligned via MAFFT 7.5 (Kuraku et al., 2013). To highlight protein regions of greater weights, we represent the multiple sequence alignments as matrices where the proteins are replaced by their corresponding vectors and plotted as heatmaps. The gaps were filled with zeros. Finally, InterproScan 5.0 [6], [15] was applied to annotate important protein motifs, and Shannon entropy was calculated for each position to reveal conserved regions. For motif analyses, we chose the proteins used as a cluster reference by CD-HIT.

**Algorithm 1:**
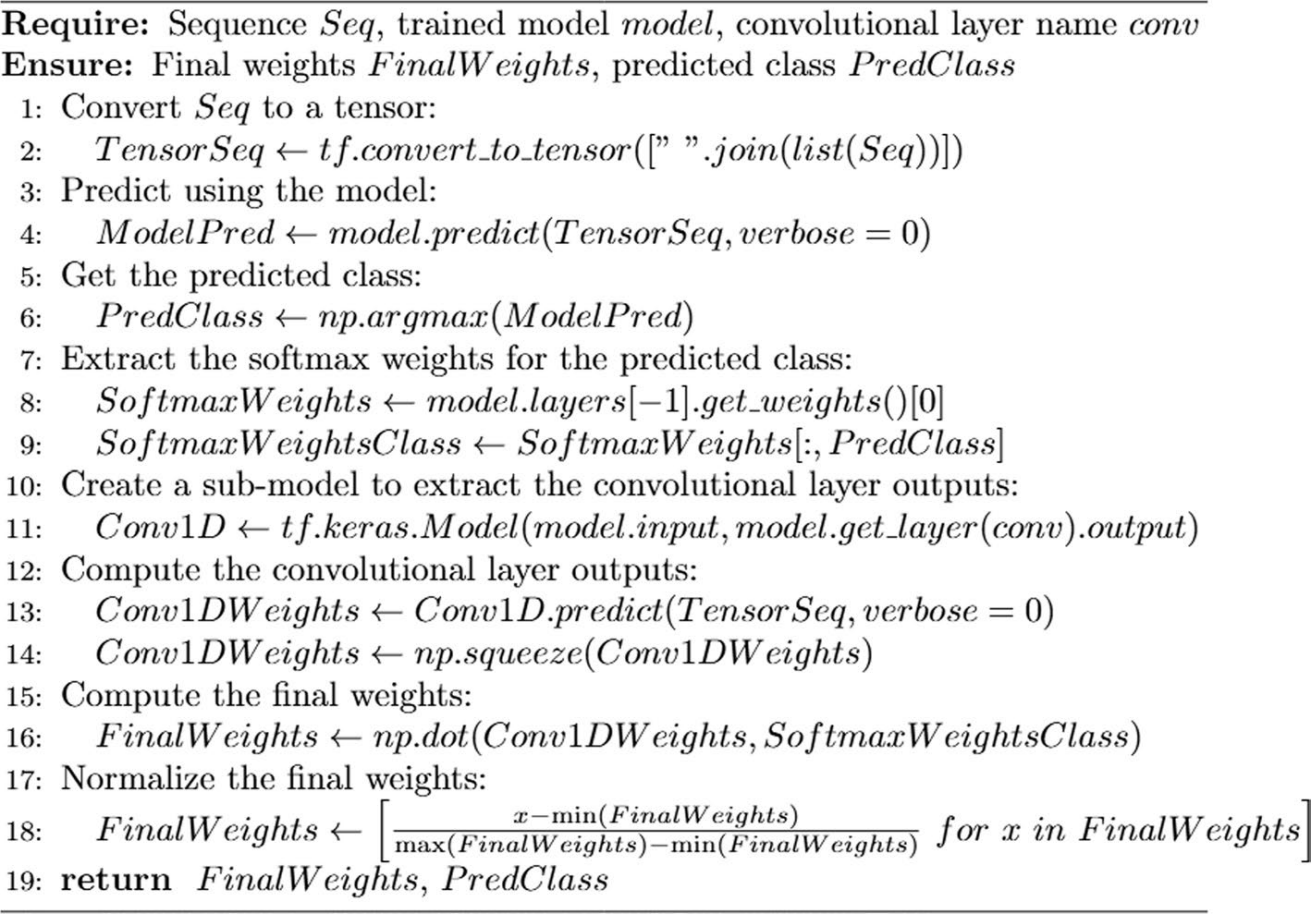
Extract weights

### CNN cluster ability

To assess cluster ability, we leveraged the information embedded in the global average pooling layer. As this layer works as a bridge between feature extraction and the final classification made by the dense layer, its internal state represents each protein imputed as a multidimensional vector with summarized features extracted upstream. Therefore, we designed an algorithm (Algorithm 2) that removes the dense layer and makes the model output the global average pooling vectors.

**Algorithm 2:**
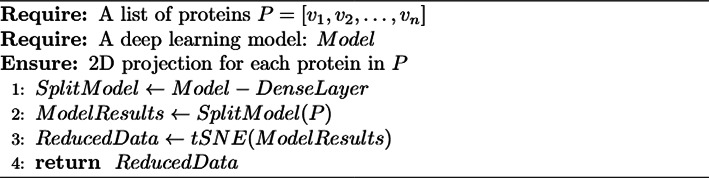
Dimension reduction

Algorithm 2 was applied to the holdout test set since the model has no knowledge of these proteins and the resulting vectors (Additional File 1; Supplementary Figure 1). The resulting vectors were then concatenated into a matrix and subsequently reduced to two dimensions through t-distributed stochastic neighbor (t-SNE) from scikit-learn 1.4.2 with the following parameters: t-SNE learning rate set to *auto*, iterations = 1000, and perplexity = 50. The original index order from the holdout test was kept. Thus, the reduced matrix contained a projection to two dimensions of the summarized features from our model, which could be associated with the respective classes of the proteins.

### Evaluation metrics

To address biases stemming from uneven data, we incorporated class weights to avoid errors in model performance assessment linked to an imbalance in protein class sizes. The calculation of precision (Eq. 1), recall (Eq. 2), the F1-score (Eq. 3), and the categorical cross-entropy error (Eq. 4). For dealing with class imbalance, class weights were considered directly in the loss function during training and indirectly by scikit-learn metrics during the evaluation.

1 $$ = \frac{{{\text{TP}}}}{{{\text{TP}} + {\text{EP}}}} $$

2 $$ {\text{Recall}} = \frac{{{\text{TP}}}}{{{\text{TP}} + {\text{EP}}}} $$

3 $$ {\text{F1 - score}}\,{ = }\,{2} \cdot \frac{{{\text{Precision}}\, \cdot \,{\text{Recall}}}}{{{\text{Precision}}\, + \,{\text{Recall}}}}, $$

4 $$ W{\text{eightes}}\,{\text{Categorical}}\,{\text{Cross - Entropy}} = - \sum\limits_{c = 1}^{C} {w_{c} \cdot i_{i,c} \cdot \log (\widehat{y}_{i,c} )} $$

where:

TP is the number of true positives.

FP is the number of true positives.

TN is the number of true negatives.

FN is the number of true negatives.\

C is the number of classes

w_c_ is the weight for class c,

$\widehat{y}_{i,c}$ is the predicated probability by the neural network that sample *i* be longs to class c.

### Class weights calculation

The class weights (Additional File 2; Supplementary Table 1) were computed using scikit-learn’s compute_class_weight (version 1.4.2) (Eq. 5), which assigns weights inversely proportional to class frequencies in the dataset to address imbalance.

5 $$ w_{c} = \frac{{{\text{NS}}}}{{{\text{NC}} \times {\text{NS}}_{{\text{i}}} }} $$

where

NS: Totalnumberofsamplesinthedataset

NC: Numberofuniqueclasses

NS_i_: Numberofsamplesinclass

## Results

### Dataset curation and the lack of reference sequences

CARD and National Database of Antibiotic-Resistant Organisms (NDARO) are the two largest public databases used as sources of reference sequences for functional annotation of antimicrobial resistance proteins. Since the CARD requires experimental validation, it has been widely used in several studies [2, 22, 27], [39, 40], [43]. Even part of the NDARO database is composed of CARD. However, since a protein can be annotated only if it is highly similar to a reference in the CARD, researchers are constantly rediscovering variations of the same resistance proteins.

We investigated the CARD database updates (Additional File 1; Supplementary Figure 2A) to check the rate at which novel reference proteins were added over the last few years. Our findings revealed that, as expected, the total number of proteins reached a plateau, while antimicrobial resistance has been increasing [28] and making infection treatment ineffective [25]. The NCRD95 database extended CARD content using homology search and was, therefore, selected for this work.

Originally, NCRD95 contained 29 antimicrobial resistance protein classes (Additional File 1; Supplementary Figure 2B) from 10603 proteins in the multidrug efflux pumps class to 2 in the diaminopyrimidine class. This high degree of imbalance compromises training, as classes with few examples may not provide enough information for generalization (B. [39, 40]. Therefore, we plotted a cumulative curve (Additional File 1,Supplementary Figure 2C) and selected the 10 most abundant classes (Additional File 1; Supplementary Figure 2D), as beyond this point, more classes did not substantially increase the total number of proteins. For instance, increasing protein classes from 10 to 15 would add only 1103 proteins (a 3% increase) to our final database. We also performed the same process for each class individually to eliminate less representative subclasses and ensure internal homogeneity.

Data leakage among classes (i.e., classes that share information) that involve multidrug resistance proteins has also been addressed. The multidrug class consists mainly of efflux pumps that extrude different antimicrobial molecules from bacterial cells, which means that the same protein may be classified into two classes depending on the experimental design. For example, we found RND efflux pumps in the multidrug, beta-lactam, and macrolide classes. Therefore, we removed the multidrug class and all efflux pumps found in our dataset, regardless of the protein class. Finally, we constructed a homology-based dataset composed of nine antimicrobial resistance proteins plus one class of non-resistance proteins (Additional File 1; Supplementary Figure 1D).

### Deep learning model evaluation

The Keras Hyperband algorithm took 83 rounds of heuristic search to optimize the set of hyperparameters, which yielded a CNN model with the lowest loss value possible (Additional file 2; Supplementary Table 2). However, low loss values must be taken cautiously in deep learning research since neural networks can learn the intrinsic noise of the training set rather than the meaningful signal. In the protein research field, this may occur when there are copies of proteins shared by training and test sets, so the model would recognize idiosyncratic features instead of general features. We mitigated the risk of overfitting resulting from data leakage by restricting the sequence similarity between the training and test sets to a maximum of 95%.

Another precaution taken was the split point that created the training set. Although randomized, the process may lead to a biased model if the proportion of classes between training and test is not respected. We addressed this issue by cross-validating our model with 5 stratified sub-splits of our training set. The convergence curves of cross-validation (Additional File 1; Supplementary Figure 3A), the final model training metrics (Additional File 1; Supplementary Figure 3B and C), and evaluation metrics (Table 2) on the holdout test set did not show clear trends of overfitting regardless of the training subset.

**Table 2:** Cross-validation results

Class	Mean precision	Mean recall	Mean F1-score	Proteins
MLS	0.96 $\pm$ 0.05	0.95 $\pm$ 0.02	0.95 $\pm$ 0.02	554
NonR	0.83 $\pm$ 0.02	0.97 $\pm$ 0.02	0.89 $\pm$ 0.01	4655
Aminoglycoside	0.96 $\pm$ 0.03	0.95 $\pm$ 0.01	0.96 $\pm$ 0.02	1199
Beta-lactam	0.99 $\pm$ 0.01	0.96 $\pm$ 0.01	0.97 $\pm$ 0.01	1844
Chloramphenicol	0.99 $\pm$ 0.01	0.98 $\pm$ 0.01	0.99 $\pm$ 0.01	423
Glycopeptide	0.99 $\pm$ 0.01	0.99 $\pm$ 0.04	0.99 $\pm$ 0.002	5922
Macrolide	0.98 $\pm$ 0.01	0.87 $\pm$ 0.07	0.92 $\pm$ 0.04	168
Phosphonic Acid	1.0	0.99 $\pm$ 0.03	0.99 $\pm$ 0.015	687
Rifamycin	0.99 $\pm$ 0.01	0.98 $\pm$ 0.01	0.99 $\pm$ 0.003	570
Tetracycline	0.98 $\pm$ 0.03	0.98 $\pm$ 0.01	0.98 $\pm$ 0.01	1070

We use the holdout test set to compare deep learning and protein alignment on antimicrobial resistance protein annotation. To represent the alignment-based approaches, the state-of-the-art tools Resistance Gene Identifier (RGI) 6.0.3 [1] and AMRFinderPlus 4.0.19 [10] were selected. On the deep learning approach, our CNN and the fine-tuned ESM2. Since RGI and AMRFinderPlus do not report Non-resistant proteins, to fairly benchmark them, we equalized the number of classes in their outputs by considering as NonR the proteins not classified as resistant by RGI or AMRFinderPlus.

**Fig. 1:**
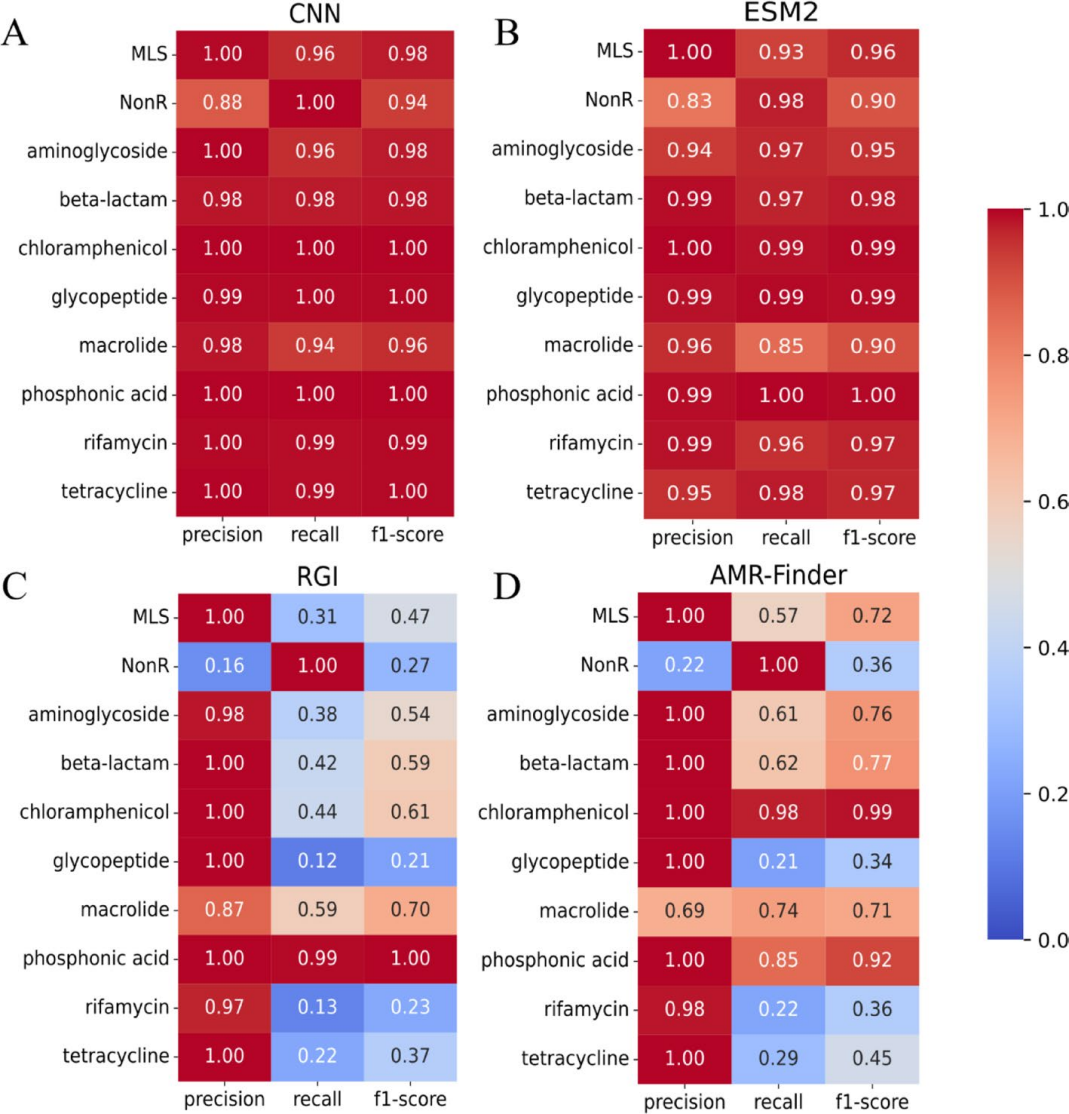
Classification reports. Benchmarking of CNN (**A**), ESM2 (**B**), RGI (**C**), and AMR-Finder (**D**) on the holdout test set. The metrics were calculated with class weight values to avoid bias due to class imbalance

The deep-learning-based approach (Figure 1A and B) presented high values of recall (>0.95) across antimicrobial resistance protein classes, although their performance slightly decreased in the precision of the nonresistance classes. On the other hand, alignment-based tools (Figure 1C and D) only achieved comparable values of precision and recall in cases where there are sequences that serve as references for annotating proteins.

**Fig. 2:**
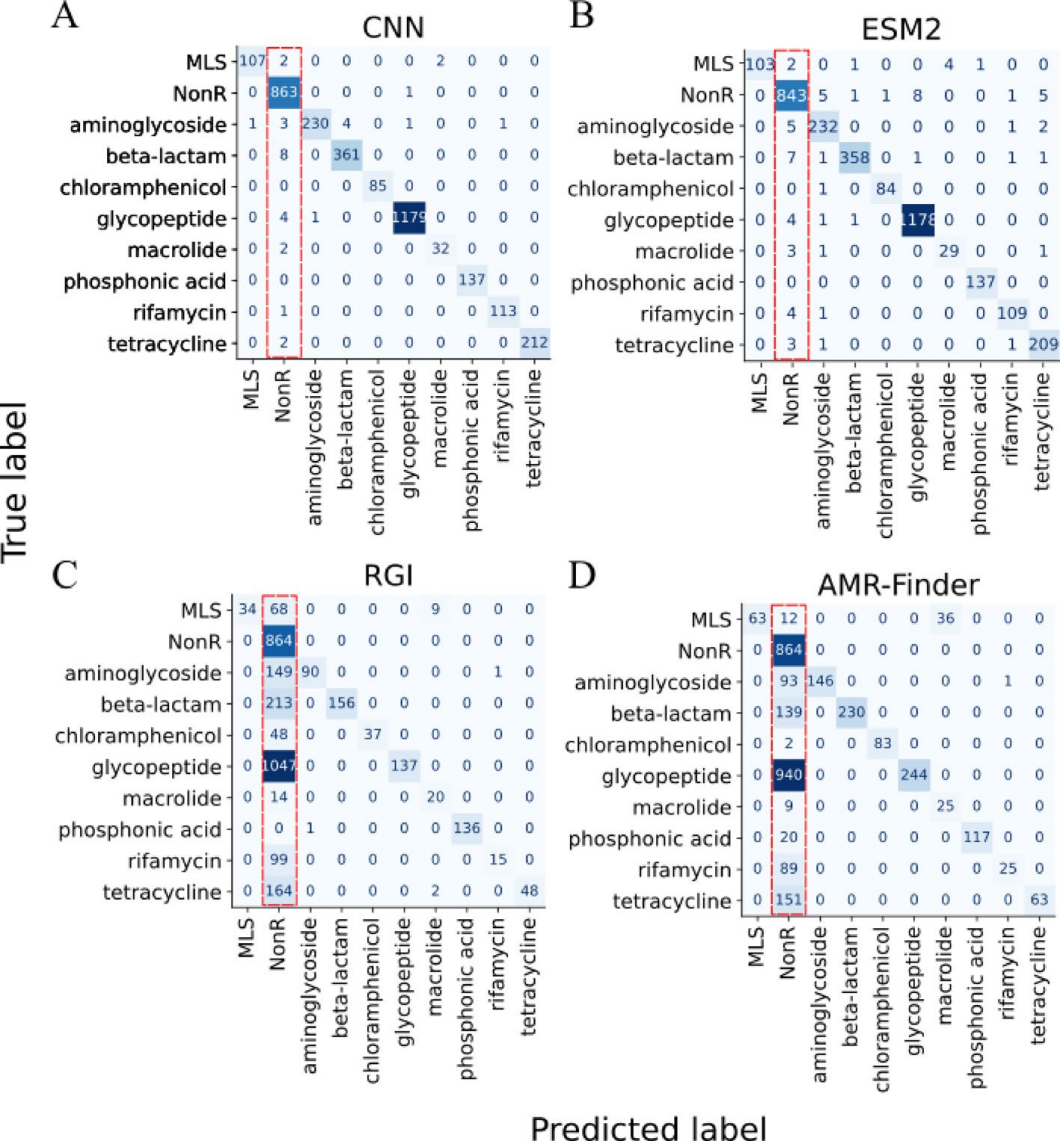
Confusion matrices of CNN (**A**), ESM2 (**B**), RGI (**C**), and AMR-Finder (**D**) on the holdout test set. The red-dashed squares highlight the false negative results of each benchmarked tool

Confusion matrices showed in detail the misclassifications of each tool in our benchmarking. CNN and ESM2 (Figure 2A and B) presented a few antimicrobial resistance proteins annotated as non-resistance proteins. RGI and AMR-FinderPlus (Figure 2C and D), on the other hand, presented more false negative results, mostly in the glycopeptide class.

### Generalization in out-of-distribution data

Antimicrobial resistance proteins are limited in number due to the sequence alignment drawbacks mentioned earlier. For instance, CTX-M beta-lactamases share 94% of amino acid identity [38]. Such a lack of diversity may impose biases into deep learning models, which would perform well only on proteins identical to their training set but poorly on dissimilar proteins [3]. Therefore, we investigated the limits of generalization of our CNN regarding the sequence similarity using our holdout test set and the NDARO.

DIAMOND alignments showed that approximately 68% (2292 out of 3352 proteins) of the holdout test set aligned to the training set with an identity range of 29% to 95%. This indicates that our CNN is not limited to identical variants of the training set; otherwise, the model would demonstrate a significant decrease in its performance. CNN’s performance on the remaining 32% (1060 proteins) corroborates this hypothesis, as the values of precision, recall, and f1-score remained high (Additional File 1; Supplementary Figure 4A). In fact, for chloramphenicol and phosphonic acid, they remained equal to one. The decrease observed in the precision of non-resistant proteins resulted from the few false negatives from the other classes.

Our CNN yielded similar results on NDARO proteins. From the 5959 resistant proteins in it, 5726 aligned to our training set, and 98% of them (5654) were correctly classified despite their similarity range to our training set being from 30 to 100% (Additional File 1; Supplementary Figure 4B). From the set of proteins that did not align (233 in total), DeepSEA misclassified 42 proteins from beta-lactam (Additional File 1; Supplementary Figure 4C). A manual inspection revealed that the majorly of these 42 proteins are subclasses absent in our training set, such as VEB (n=11), CFX (n=12), and MUN (n=6) beta-lactamases (Additional File 1; Supplementary Figure 4D). Indeed, only the subclass OXA (n=1) contains examples in our training and was wrongly assigned as non-resistant. Together, these results provided evidence of true generalization as our CNN annotated out-of-identity-distribution proteins, as long as there are examples of that protein class in the training set, even with low sequence similarity.

### Model explainability

**Fig. 3:**
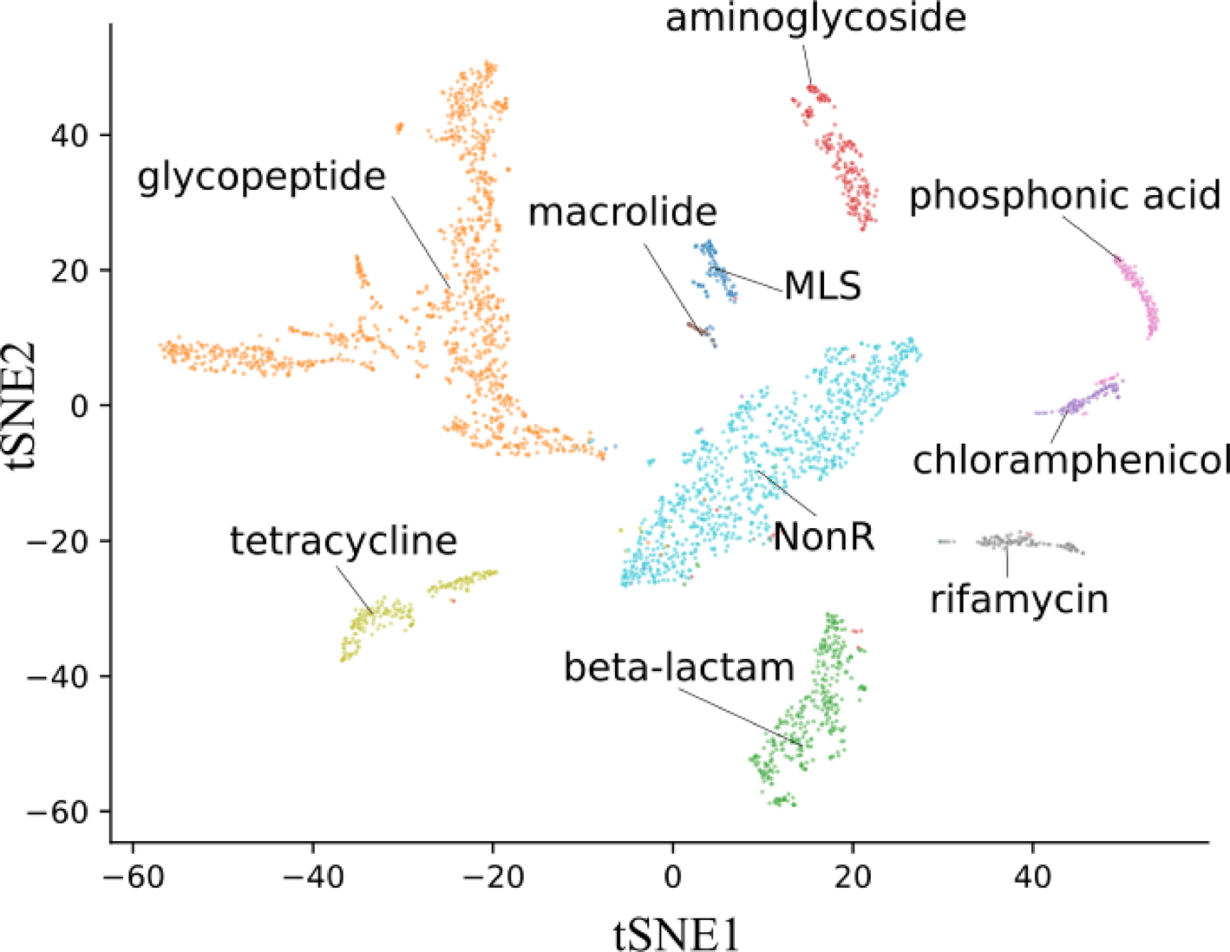
CNN class clusters. The t-SNE plot is made of the reduced weight matrix retrieved from the global average pooling layer as described in Algorithm 2 (see Methods)

The results exposed above led us to investigate the latent space produced by the feature extraction block to reveal which protein features the CNN learned to yield such high performance. The global average pooling layer summarizes the features extracted by the convolutional layers. Therefore, we applied Algorithm 2 to make the model return these vectors instead of classifying proteins and reducing them with t-SNE. Our CNN separated the holdout test set according to protein classes (Fig. 3) with a few misclassifications, indicating that the model successfully differentiated sets of weights for each protein class, which shows the model’s ability to distinguish resistant from non-resistant proteins and predict the correct resistance classes.

As these results indicate that there are diagnostic features learnt from each protein class, we searched for them in the feature matrices produced by the convolutional layers and investigated if they could be associated with biological functions. Briefly, Algorithm 1 highlights important features by dot-producting the weights from the dense layer and the feature matrix for a given imputed protein. Pairing the resulting vector with the original protein sequence reveals residues of importance. Also, we overlapped the feature vectors and protein domains annotated by InterproScan 5 to add biological meaning.

**Fig. 4:**
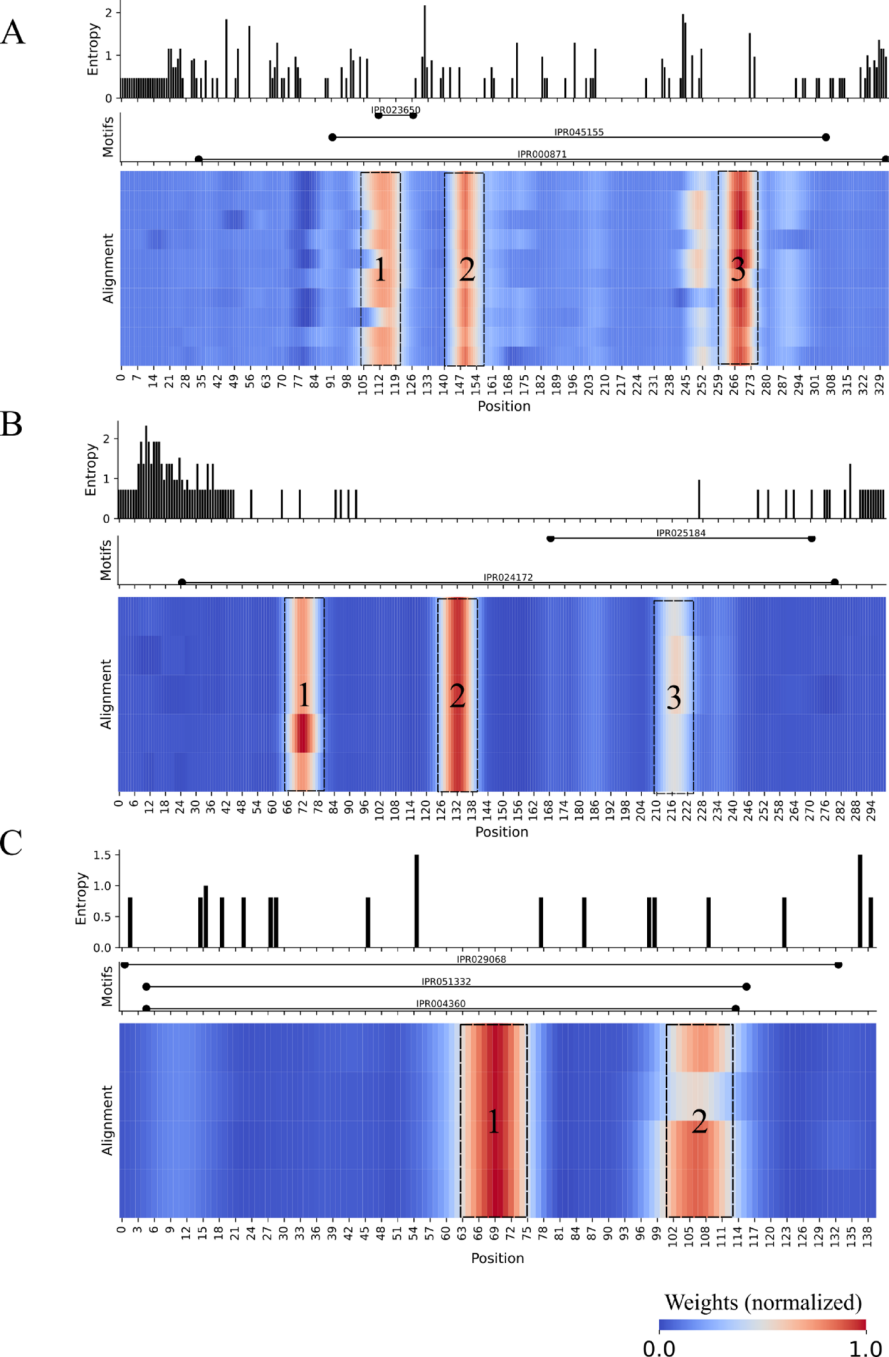
CNN neurons firing patterns for resistance proteins from the beta-lactam (**A**), aminoglycoside (**B**), and phosphonic acid (**C**) classes. Each plot is divided into a bar plot with Shannon entropy values, a line plot with protein domain extensions found by Interproscan 5, and the CNN weights spread over a multiple sequence alignment of a cluster of proteins selected by CD-HIT (see Methods). The black-dashed squares highlight the most important regions (hotspots) for classification

Our results showed that CNN selected conserved regions (determined from multiple sequence alignments) as information hotspots for classification. For instance, class-A beta-lactamases (Fig. 4A) alignment presented three main regions where CNN neurons fired to predict this class of enzymes, all of which are within the large beta-lactamase signature (IPR000871). The first hotspot contains the class-A active site (IPR023650) where the catalytic residues (S–K) are located [13], [16]. The last hotspot includes the binding site GTK, another conserved and diagnostic subdomain of class-A beta-lactamases.

The aminoglycoside-modifying enzyme AadA alignment (Fig. 4B) also has three important conserved regions that belong to the adenylyltransferase AadA/Aad9 domain (IPR024172). The last hotspot is in the adenylyltransferase AadA, C-terminal domain (IPR025184), and the first belongs to the nucleotidyltransferase domain from the superfamily of proteins in which adenylyltransferases are included. Fosfomycin thiol transferase (FosA), the example of the phosphonic acid (or fosfomycin) class (Fig. 4C), has two hotspots in conserved domains related to fosfomycin resistance: fosfomycin resistance protein (IPR051332), glyoxalase/fosfomycin resistance/dioxygenase domain (IPR004360), and glyoxalase/bleomycin resistance protein/dihydroxybiphenyl dioxygenase (IPR029068).

**Fig. 5:**
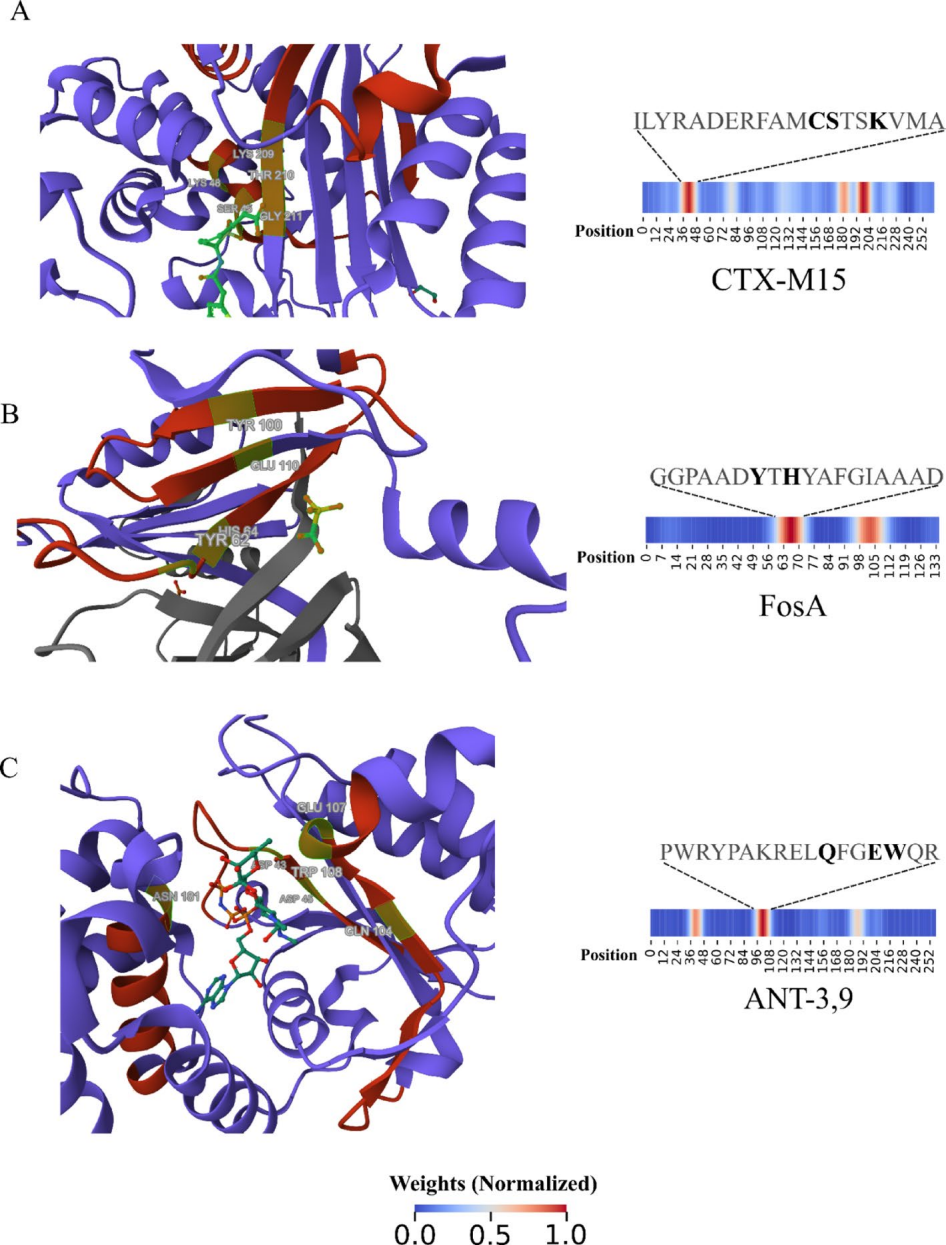
CNN model interpretability. PDB 3D structures of a CTX-M beta-lactamase (**A**), a fosfomycin thiol transferase (**B**), and an aminoglycoside nucleotidyltransferase (**C**) colored according to CNN neuron firing patterns. The green-highlighted residues on the structures and the black-highlighted above heatmaps are annotated as binding sites to antimicrobial molecules. The grey structure on B is the other fosfomycin thiol transferase monomer

Given these results, we enquired whether our model captured information from protein regions related to antimicrobial molecule inactivation in the cases of aminoglycoside and fosfomycin-resistant proteins. To investigate this, we selected enzymes in complex with antimicrobials from the Protein Data Bank (PDB) (Table 3), applied the same feature extraction method, and compared our results with PDB functional annotations. CTX-M15 beta-lactamase (PDB: 7TI0) (Fig. 5A) hotspots contain residues annotated as binding sites. Specifically, the residues S–K in which the serine (S45) residue nucleophilically attacks the lactam ring and initiates the structural modification that leads to beta-lactam molecule inactivation [5, 13, 17, 26]. Additionally, the last hotspot contains the residues K209, T210, and G211, which bind and place the beta-lactam molecule in the catalytic pocket [26].

**Table 3:** Proteins from PDB and subsequences of high importance for classification

Protein	Class	PDB ID	Hotspots
FosA	Phosphonic acid	1NKI	1: GGPAADYTHYAFGIAAAD 2: GDSFYFLDPDGHRLE
ANT3,9	Aminoglycoside	7UY4	1: GGLKPHSDIDLLV 2: PWRYPAKRELQFGEWQR 3: NVVLTLSRIWYSAV
CTX-M-15	Beta-lactam	7TI0	1: ILYRADERFAMCSTSKVMA 2: DSQRAQLVTWM 3: AASIQAGLPASWVVGDKTG

For the FosA (PDB: 1NKI) protein (Fig. 5B), our model highlighted two hotspots that build the catalyst site where the residues H64, Y100, and E110 bind the fosfomycin molecule and Y62, which bridges the active site and the dimer interface loop [19]. Similarly, the aminoglycoside (3″) (9) adenylyltransferase (PDB: 7UY4) (Fig. 5C) presented residues D43, D45, Q104, E107, W108, and D181 annotated as binding sites for spectinomycin antimicrobial (Kanchugal P and Selmer, [18]. These results confirmed that our model learned biological features from proteins without reference sequences or sequence alignment comparisons.

We checked our model’s misclassifications and found evidence of misannotated proteins in the NCRD95 dataset that were correctly classified by our CNN. In our test, the protein NR_MCR0138297.1 is labeled as aminoglycoside resistance and was classified as beta-lactam. BLASTP revealed that NR_MCR0138297.1 is 86.6% identical to a PAC-1 (NR_ABP88743.1) beta-lactamase in our training set. The same was observed for NR_WP_193096997.1, a member of the aminoglycoside resistance class that is 52.1% similar to Arr-1 (NR_PRC48666.1), a rifamycin-resistant protein.

A third example of misannotation from NCRD95 is the oleD protein, a glycosyltransferase that confers resistance only to macrolide antimicrobials [7], [33]. In this case, the protein NR_WP_166002910.1 in the training set and the NR_PZH16762.1 protein in the test set were both labeled as MLS resistance class and shared 82.8% of protein identity. However, our CNN classified NR_WP_166002910.1 as a macrolide-resistant protein.

### DeepSEA tool

To make our model available for the scientific community, the CNN was wrapped in a command-line interface tool available on https://github.com/computational-chemical-biology/DeepSEA-project. There are two usage options for DeepSEA: for resistance classification, the user can provide a raw FASTA file as input and obtain a table format file with model predictions and their respective probabilities; or for motif detection, a multisequence alignment FASTA file can be analyzed by DeepSEA, returning a heatmap with the hotspots for each aligned sequence.

## Discussion

In this work, we developed an alignment-free tool that functionally annotates proteins across multiple antimicrobial resistance classes and distinguishes resistance from non-resistance proteins without protein alignment. The DeepSEA model was trained to recognize motifs (hotspots) from resistance proteins and therefore provides an explanation for its predictions, which helps users from biological backgrounds to interpret the results. Benchmarking revealed that DeepSEA is superior to protein alignment when it comes to identifying fewer false negatives.

The protein alignment approach uses homology inference [32] as a shortcut from sequence to function in the classical sequence-structure-function paradigm [20]. However, even homologous groups, such as serine beta-lactamases, can vary considerably outside their active sites [16], forcing the databases to have reference sequences for variants in an attempt to mitigate the high rates of false negative results [2].

The lower recall values of RGI and AMRFinderPlus demonstrated that the lack of proper reference sequences in their databases led to several misclassifications of resistant proteins as non-resistant proteins. For instance, RGI and ARMFinderPlus classified 88% and 79% of the proteins that resist glycopeptides as non-resistant proteins, respectively. Moreover, both alignment-based tools presented misleading results for proteins related to resistance to beta-lactam antimicrobials. DeepSEA, on the other hand, had only 8 beta-lactam resistance proteins misclassified as non-resistant.

ESM2 and DeepSEA had similar performance even though they differ in the complexity of their architectures. ESM2 is a large language model with a complex transformer-based architecture, which required approximately 60 million proteins from the UniRef database (September 2021 version) to be pre-trained, whilst DeepSEA has a CNN-based architecture that allowed output interpretability without data perturbation. For example, to explain HMD-ARG [22] results, the author had to investigate the effect of *in silico* mutations in protein sites on model performance. This need for checking protein variations is computationally expensive and infeasible in most cases. The pioneer DeepARG [2] used a multilayer perceptron model, which can not offer explainability, and also needs to prefilter resistance-like proteins via DIAMOND [8] before model prediction itself.

During the benchmarking experiments, the DeepSEA precision value was lower for non-resistant than for other classes. This was expected due to the miscellaneous composition of the non-resistant class, which contains proteins with a plethora of functions. This lack of homogeneity of the non-resistant class forces the CNN neurons to fire at patterns restricted to the other classes. We extracted this information directly from the model and converted it to a human-readable format, contextualizing DeepSEA’s output to the information provided by multisequence alignment. This feature could be used in future research to combine 3D structure prediction with functional annotations.

DeepSEA was designed to address a key issue in antimicrobial resistance protein annotation: the false negative rates. We focused on pushing the frontiers of protein annotation made by sequence similarity. Therefore, DeepSEA does not contain genome assemblers and open reading frame predictors. Also, since genomic research pipelines often use annotation tools of a general purpose, such as Prokka [35], DeepSEA can be employed to reannotate proteins previously assigned as hypothetical and increase the knowledge on the diversity of proteins produced by bacteria or to screen large datasets of unannotated protein sequences predicted by other tools. DeepSEA was designed to find signatures of proteins for broad resistance classes. For instance, future work will address more specific questions and split the beta-lactam class according to the type of beta-lactam antimicrobial (i.e., cephalosporin, penem, to cite a few).

## Conclusion

In this work, we addressed the high rates of false negatives in antimicrobial resistance protein annotation by sequence alignment. It was demonstrated that a single CNN model, trained directly on protein sequences instead of MSA, achieved higher performance than alignment-based methods and produced only a few false negatives for every protein class in the training set without detectable overfitting. Moreover, we proposed an algorithm to extract and transform information from the CNN into meaningful human-readable insights about the model’s decision-making that help users from different backgrounds interpret the results. Future work could use multimodal data to expand these explanatory features from proteins to complex metabolic pathways of resistant bacteria to reveal protein-protein interactions that contribute to the resistant phenotype.

## Supplementary Information

Below is the link to the electronic supplementary material.

Supplementary Material 1

Supplementary Material 2

## Data Availability

DeepSEA datasets and models can be accessed and downloaded from the project’s repository on GitHub: https://github.com/computational-chemical-biology/DeepSEA-project. The additional resources used in this project were: National Database of Antibiotic Resistant Organisms (NDARO): https://www.ncbi.nlm.nih.gov/pathogens/antimicrobial-resistance/. Non-redundant comprehensive antibiotic resistance genes database (NCRD): https://github.com/LabHanmz/NCRD. Resistance Gene Identification docker, used in the benchmarking: https://github.com/arpcard/rgi AMRFinderPlus docker, used in the benchmarking: https://github.com/ncbi/amr/wiki/Installing-AMRFinder#docker. Evolutionary Scale Model used in the benchmarking: https://huggingface.co/facebook/esm2_t6_8M_UR50D. InterproScan5 used for motif verification: https://www.ebi.ac.uk/interpro/download/InterProScan/. CTX-M15 beta-lactamase structure accession: PDB 7TI0. FosA structure accession: PDB 1NKI. Aminoglycoside (3″) (9) adenylyltransferase structure accession: PDB 7UY4.

## Abbreviations

MSA: Multiple sequence alignment
CNN: Convolutional neural network
RGI: Resistance gene identifiers
NCRD: Non-redundant comprehensive resistance database
AMR: Antimicrobial resistance
HIV: Human immunodeficiency virus
AST: Antimicrobial susceptibility tests
PDB: Protein data bank
EMS: Evolutionary scale model
NonR: Non-resistant proteins
MLS: Macrolide, lincosamide, and streptogramin
