# Supplementary material for: DeepSEA: an alignment-free explainable approach to annotate antimicrobial resistance proteins
Source: BMC Bioinformatics. 2025 Sep 1;26:224. doi: 10.1186/s12859-025-06256-4 (PMC12403478; doi:10.1186/s12859-025-06256-4)
Supplement: Supplementary file 1 — Supplementary Material 1 [file 12859_2025_6256_MOESM1_ESM.docx]

**DeepSEA: an alignment-free explainable approach to annotate antimicrobial resistance proteins.**

Tiago Cabral Borelli^a,b,c^, Alexandre Rossi Paschoal^d,e^, Ricardo Roberto da Silva^a,b^

^a^ Computational Chemical Biology Laboratory, Department of BioMolecular Sciences, School of Pharmaceutical Sciences of Ribeirão Preto, University of São Paulo, Ribeirão Preto 14040-900, Brazil

^b^ NPPNS, Department of BioMolecular Sciences, School of Pharmaceutical Sciences of Ribeirão Preto, University of São Paulo, Ribeirão Preto, 14040-900, Brazil

^c^ Cellular and Molecular Biology Program, Department of Cellular and Molecular Biology of Ribeirão Preto, School of Medicine, University of São Paulo, Ribeirão Preto, 14049-900, Brazil

^d^ Bioinformatics and Pattern Recognition Group (Bioinfo-CP), Department of Computer Science (DACOM), The Federal University of Technology – Paraná (UTFPR), Cornélio Procópio, Brazil

^e^ Rosalind Franklin Institute, Harwell Science and Innovation Campus, Didcot, OX11 0QS, UK


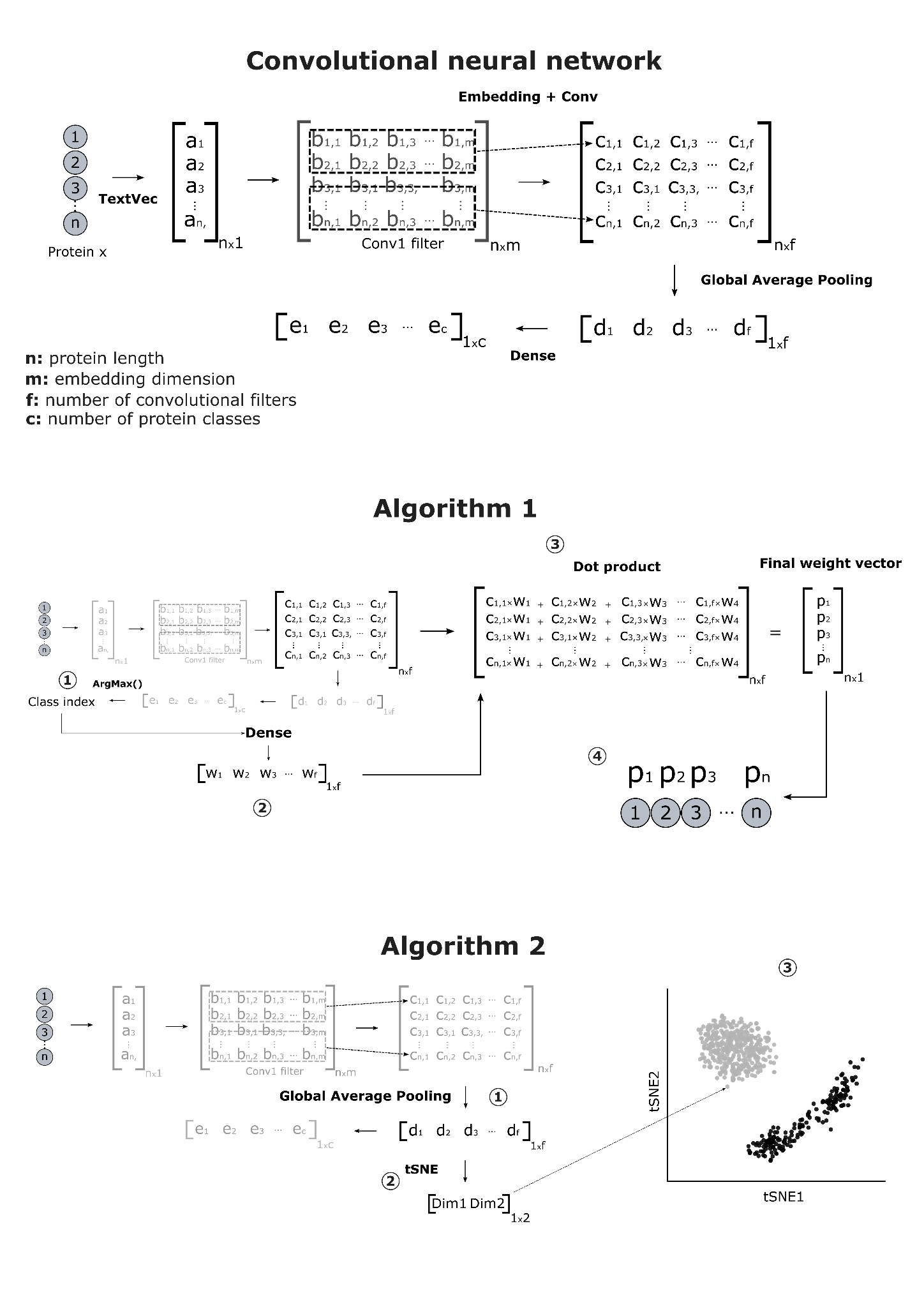
Supplementary Figure 1. Visual representation of information workflow inside a convolutional neural network (CNN) and mathematical transformation of CNN internal states by Algorithms 1 and 2 into a human-interpretable output.


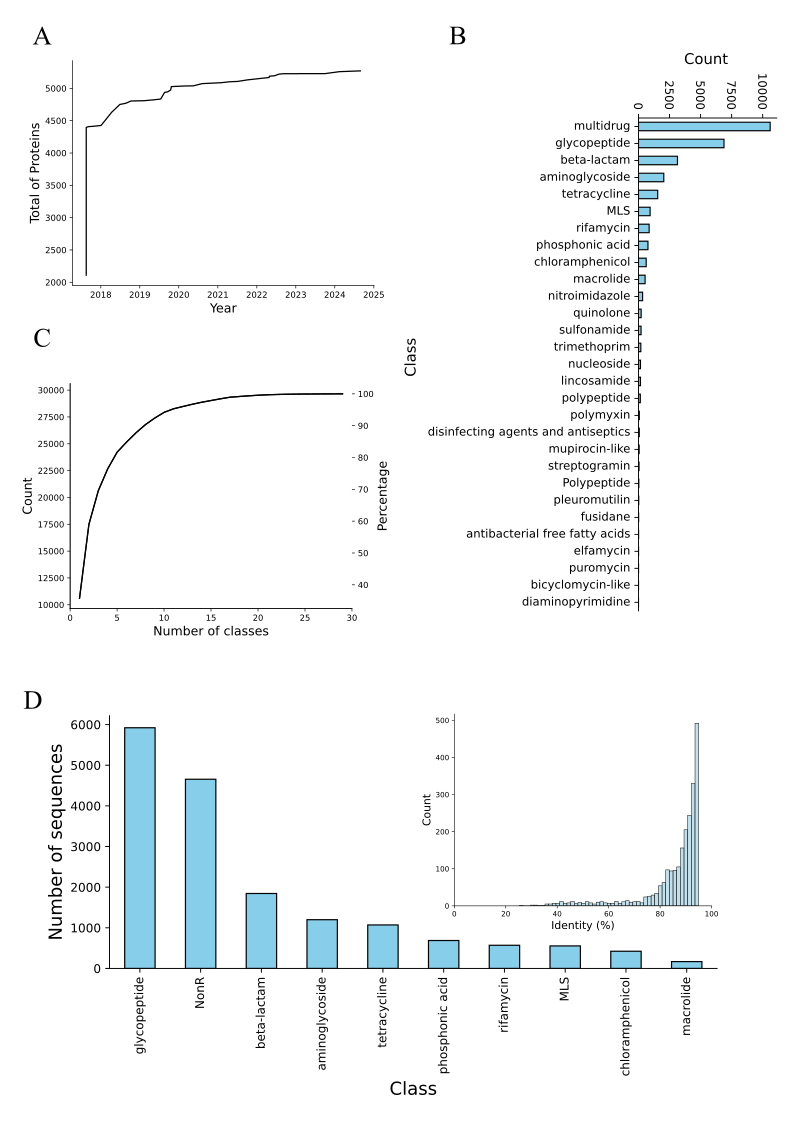


Supplementary Figure 2. Database descriptions. A) Cumulative curve of CARD database updates from early 2017 to late 2024. B) NCRD95 original class distribution. C) Cumulative curve of NCRD95 before filtering. D) The bar plot shows the resistant protein class distribution after filtering NCRD95 and NonR class insertion. This final dataset was used for developing our CNN model. Additionally, the histogram shows the protein similarity distribution between training and test sets. The alignment was performed considering the converge parameter equals 100% to avoid local alignment and e-value < 0.001.

Supplementary Figure 3. Model convergences. A) Convergence curve for the CNN model trained using the complete training set. The limited number of epochs is due to early stopping to avoid overfitting. The dashed curve represented the loss calculator using the holdout test set. B) Convergence curve for an extended period (1000 epochs) for checking overfitting. C) Converge curves from 5-fold cross-validation. The training set was randomly subdivided at five different points as detailed in Methods.
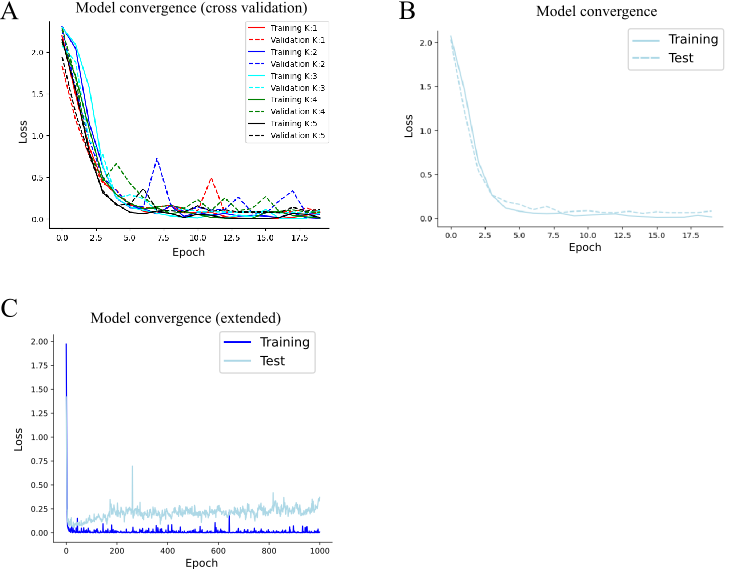


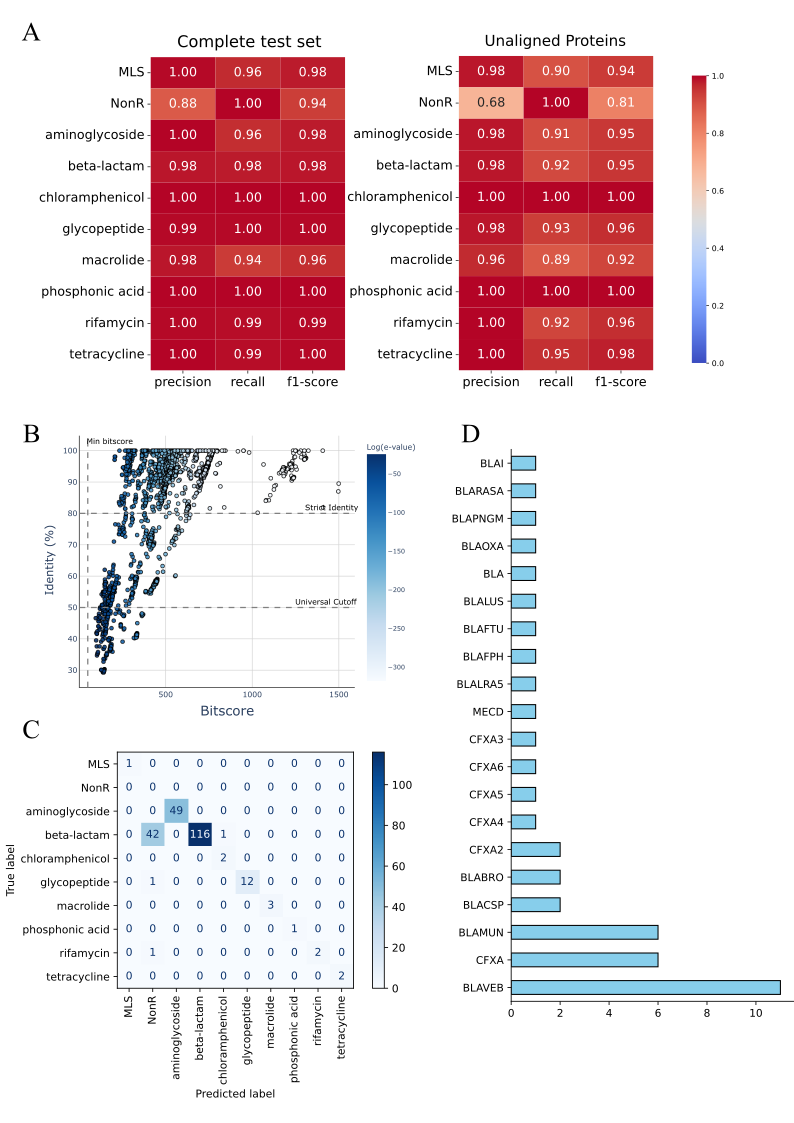


Supplementary Figure 4. The limits of generalization. A) Evaluation metrics of the entire test set (n=3352) and the subset of proteins that did not align to our training set (n=1060). B) The scatterplot contains alignment metrics of antimicrobial resistance proteins from NDARO that were correctly classified by DeepSEA (5654 out of 5959 proteins). The identity cutoff values were based on Arango’s work [(Arango-Argoty et al., 2018)](https://www.zotero.org/google-docs/?rUaNWy) and the minimum bitscore value from Pearson’s paper about protein homology [(Pearson, 2013)](https://www.zotero.org/google-docs/?oWyWCh). C) DeepSEA classification of the remaining 233 proteins that did not align to our training set. D) Count of beta-lactam misclassified as NonR by DeepSEA.
