# Supplementary material for: DeepSEA: an alignment-free explainable approach to annotate antimicrobial resistance proteins
Source: BMC Bioinformatics. 2025 Sep 1;26:224. doi: 10.1186/s12859-025-06256-4 (PMC12403478; doi:10.1186/s12859-025-06256-4)
Supplement: Supplementary file 2 — Supplementary Material 2 [file 12859_2025_6256_MOESM2_ESM.docx]

**DeepSEA: an alignment-free explainable approach to annotate antimicrobial resistance proteins.**

Tiago Cabral Borelli^a,b,c^, Alexandre Rossi Paschoal^d,e^, Ricardo Roberto da Silva^a,b^

^a^ Computational Chemical Biology Laboratory, Department of BioMolecular Sciences, School of Pharmaceutical Sciences of Ribeirão Preto, University of São Paulo, Ribeirão Preto 14040-900, Brazil

^b^ NPPNS, Department of BioMolecular Sciences, School of Pharmaceutical Sciences of Ribeirão Preto, University of São Paulo, Ribeirão Preto, 14040-900, Brazil

^c^ Cellular and Molecular Biology Program, Department of Cellular and Molecular Biology of Ribeirão Preto, School of Medicine, University of São Paulo, Ribeirão Preto, 14049-900, Brazil

^d^ Bioinformatics and Pattern Recognition Group (Bioinfo-CP), Department of Computer Science (DACOM), The Federal University of Technology – Paraná (UTFPR), Cornélio Procópio, Brazil

^e^ Rosalind Franklin Institute, Harwell Science and Innovation Campus, Didcot, OX11 0QS, UK

Supplementary Table 1. Class weights and proportions

| **Class** | **Proportion in the dataset (%)** | **Weight** |
| --- | --- | --- |
| MLS | 3 | 3.03 |
| NonR | 26 | 0.39 |
| Aminoglycoside | 7 | 1.40 |
| beta-lactam | 11 | 0.91 |
| chloramphenicol | 3 | 3.96 |
| glycopeptide | 35 | 0.28 |
| macrolide | 1 | 9.98 |
| phosphonic acid | 4 | 2.44 |
| rifamycin | 3 | 2.94 |
| tetracycline | 6 | 1.57 |

Supplementary Table 2. CNN detailed architecture.

| **Layer** | **Hyperparameters** |
| --- | --- |
| TextVectorization | max tokens = 20  output_sequence_length = 1024 |
| Embedding | input dim = 20  output dim = 50 |
| Conv1D | filters (kernels) = 968  kernel size = 9  activation = relu  padding = same |
| Dropout | rate = 0.25  seed = 42 |
| Conv1D | filters (kernels) = 464  kernel size = 9  activation = relu  padding = same |
| Dropout | rate = 0.25  seed = 42 |
| Conv1D | filters (kernels) = 296  kernel size = 9  activation = relu  padding = same |
| Dropout | rate = 0.25  seed = 42 |
| Conv1D | filters (kernels) = 520  kernel size = 9  activation = relu  padding = same |
| GlobalAveragePooling1D |  |
| Dense | neurons = 10  activation = softmax |
